# A Case-Control Study of Dietary Choline Intake and Risk of Colorectal Cancer Modified by Dietary B-Vitamin Intake

**DOI:** 10.3390/nu16234200

**Published:** 2024-12-05

**Authors:** Alyssa Y. Chen, Eryn K. Matich, Jonathan Laryea, Ping-Ching Hsu, Lihchyun Joseph Su

**Affiliations:** 1 School of Medicine, The University of Texas Southwestern Medical Center, Dallas, TX 75390, USA; 2 School of Public Health, The University of Texas Houston Health Science Center at Houston, Houston, TX 77030, USA; 3 Department of Environmental Health Sciences, Fay W. Boozman College of Public Health, University of Arkansas for Medical Sciences, Little Rock, AR 72205, USAphsu@uams.edu (P.-C.H.); 4 College of Medicine, University of Arkansas for Medical Sciences, Little Rock, AR 72205, USA; 5 Peter O’Donnell School of Public Health, The University of Texas Southwestern Medical Center, Dallas, TX 75390, USA

**Keywords:** choline, B vitamins, colorectal cancer

## Abstract

Background/Objectives: The incidence of colorectal cancer (CRC) is rising, and Western diets high in red and processed meats may be contributing. It is important to identify dietary nutrients that increase CRC risk and perhaps interventions that may modulate such risk. The relationship between dietary choline intake and CRC is still unclear. We hypothesize that high dietary choline intake is associated with greater CRC risk, and B vitamin supplementation may modify this risk. Methods: In this case-control study, we collected demographic and dietary data using the validated National Cancer Institute CRC Risk Assessment Tool and Dietary Health Questionnaire III and analyzed colonoscopy outcomes. Logistic regression and stratified analyses were performed to calculate adjusted odds ratios and evaluate for effect modification. Results: Of 52 total patients, 21 had a normal colonoscopy result, and 31 were found to either have benign polyps or CRC. The average dietary choline intake was 207 mg/day in the normal group and 297 mg/day in the abnormal outcome group. A doubling in dietary choline intake was significantly associated with increased odds of polyps or CRC (OR 25.32, 95% CI 1.95–327.94). When stratified by vitamin B levels, the effect modification was difficult to confidently quantify due to the limited sample size. Conclusions: Our findings suggest that higher dietary choline intake may be associated with an increased risk of CRC and its precursors, such as polyps. Although the potential modifying role of B vitamins was inconclusive, this study underscores the need for larger-scale research to further explore these associations and to assess the potential of dietary interventions in reducing CRC risk.

## 1. Introduction

Colorectal cancer (CRC) is the third leading cause of death from cancer worldwide. Once known to be a disease affecting adults older than 50, the incidence of CRC is rising in younger adults at an alarming rate. The role of diet is a well-documented risk factor for CRC. Other risk factors include smoking, family history, alcohol use, sedentary lifestyle, etc. As the Western diet is heavily processed and high in red meats, diet may be contributing to the rising incidence in our younger adults. Thus, it is imperative that we not only better identify dietary nutrients associated with CRC risk but also identify dietary interventions that may reduce or modulate the deleterious effects of such dietary nutrients.

A diet high in red and processed meats and in fat content is well understood to be deleterious in promoting the development of CRC [1,2]. Choline and its oxidative product, betaine, are abundantly present in animal products high in fat, such as red and processed meats [3,4]. Though the exact mechanisms are still being uncovered, there is growing evidence in the role of our gut microbiota in modulating such high-risk diets and their contributions to colorectal carcinogenesis [5]. One such microbial metabolic byproduct that has recently been implicated is trimethylamine N-oxide (TMAO), which is derived from precursor nutrients such as choline and betaine [6,7]. TMAO has been shown to be pro-inflammatory, promote angiogenesis, and induce colorectal cancer cell proliferation [8].

Few studies have investigated the association between dietary choline intake and CRC, but the results so far have been conflicting [9,10,11]. The first epidemiologic study was done in 2008 on women in the United States [11]. This study found a positive association between choline intake and colorectal adenomas. A 2010 study on men in the United States found no significant association between dietary choline intake and CRC [12]. Another study done in 2014 on postmenopausal women in the United States found that plasma choline was insignificantly positively associated with CRC [10]. More recently, in 2015, a large-scale case-control study in a Chinese population concluded that higher intake of dietary choline was protective against CRC [9].

Many of these prior studies focused on the role of choline in one-carbon metabolism and hypothesized that higher choline levels would be protective against CRC and colorectal polyps. Like most metabolites in our complex metabolic physiology, choline is involved in multiple pathways. One such pathway is the role of choline as a precursor to gut microbiota-mediated formation of TMAO, as mentioned previously [6,7]. Additionally, choline and betaine are intimately involved in one-carbon metabolism, which is required for DNA synthesis, methylation, and stabilization [4,10]. The complex one-carbon metabolism requires B vitamins, such as B2, B6, B9, and B12, to act as cofactors for the enzymes mediating the reactions [13]. The pathway that metabolites like choline ultimately partake in depends on a myriad of factors, chief among them the concentration and availability of other substrates involved in the pathways. Based on Le Chatlier’s principle of equilibrium, the concentration of B vitamins may determine whether choline is used for 1-carbon metabolism for DNA synthesis or in the gut for microbial production of TMAO.

We hypothesize that if one consumes a diet that is deficient in B vitamins, a diet rich in choline will preferentially act as precursors in the production of pro-inflammatory TMAO, increasing the risk for CRC. We hope the inverse is also true, that diets appropriately supplemented with B vitamins can modulate the harmful risk of diets high in choline. Studies have supported this hypothesis in that the association of plasma levels of TMAO and CRC risk was restricted to patients with low serum B12 levels [10]. In this single-center case-control study, we analyze dietary patterns and colonoscopy results to better understand the association between dietary choline intake and CRC and colorectal polyp formation and to test the hypothesis that dietary B-vitamin levels may modify the relationship.

## 2. Materials and Methods

### 2.1. Data Collection

**Figure 1:**
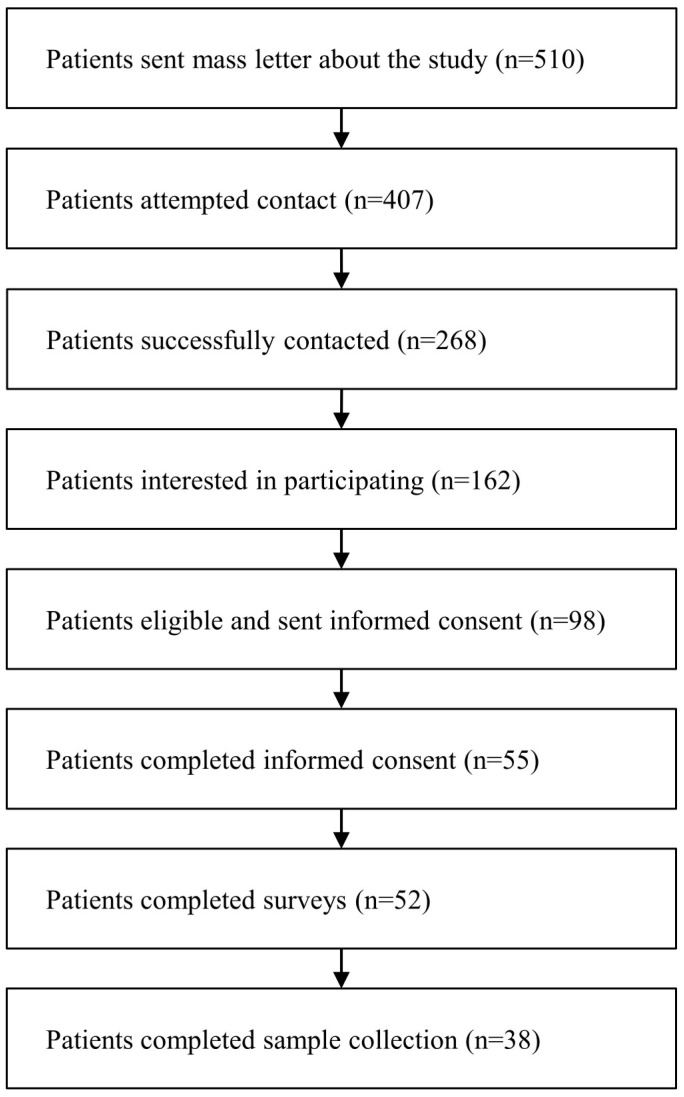
Subject recruitment flowchart.

This is a single-center, case-control study. The dataset used for this study was designed, obtained, and shared by collaborators at the University of Arkansas Medical Sciences (UAMS). The study was approved by the Human Subjects Committee at UAMS (Protocol 239622). Patients who had received a colonoscopy at UAMS from 1 January 2019 through 31 December 2021, were eligible for inclusion. Patients were ineligible if they had previously received any cancer treatment. Patients were selectively recruited using a quota sampling framework to ensure that participants were evenly distributed based on racial ethnicity, gender/sex, and health insurance status. The recruitment process is summarized in Figure 1. Digital surveys were completed prior to sample collection. The survey questions were modified from validated instruments, such as the National Cancer Institute (NCI) CRC Risk Assessment Tool [14] and the NCI Diet History Questionnaire (DHQ) III [15,16]. The Diet*Calc software [17] was used to analyze the DHQ data to generate nutrient and food group intake reports. Our primary outcome is the colonoscopy result, categorized as normal or abnormal. Normal was defined as a normal colonoscopy result. Abnormal was defined as CRC or polyps identified on colonoscopy. Of note, the polyps for which biopsy results were available were mostly tubular adenomas without additional data on grade, size, or number to further risk stratify.

### 2.2. Data Cleaning

**Figure 2:**
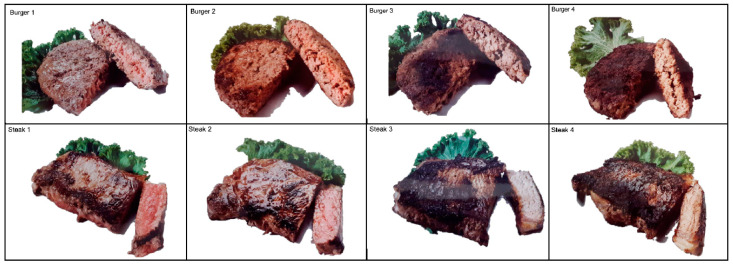
Meat doneness visual scale included in questionnaire.

To study each feature in a reproducible manner, we describe our feature representation. Smoking was categorized as a nonsmoker, past smoker, or current smoker. Exercise was represented as the minutes per week of walking, lifting, light activity, moderate activity, or vigorous activity. The minimum number of the provided survey range was used in the calculation. The DHQ questionnaire results were used to calculate the alcohol, folate, B-vitamin, choline, betaine, and cured meat protein levels. Processed meat intake was defined as the DHQ cured meat protein levels. Meat doneness was measured via a visual scale, as shown in Figure 2, with each pictorial corresponding to a number. The average meat doneness across burgers and steaks was used. For survey responses that were left blank or answered as “Don’t know or decline to respond”, values were considered null. Due to the limited sample size, null values were imputed with the mean to preserve sample sizes and minimize the variance of imputed features. To ensure that mean imputation did not affect our results, the analysis was repeated by excluding patients with missing values.

### 2.3. Statistics

The mean and standard deviation were reported for continuous variables. Frequency and percentage were reported for categorical variables. Continuous variables were tested for normality visually and objectively based on D’Agostino and Pearson’s test [18,19]. Features that did not follow a normal distribution were log-transformed. Base 2 was used for ease of interpretation of odds ratio results. Differences in baseline characteristics were then assessed parametrically by two-sample T-tests for continuous variables and chi-square tests and Fisher’s exact test for categorical variables where appropriate [20]. Spearman correlation coefficients were calculated to confirm the relationship between meat intake variables and calculated dietary choline intake. Logistic regression modeling was performed to evaluate the relationship between dietary choline intake and abnormal colonoscopy result. To adjust for other known risk factors of CRC, we included age, sex, smoking, family history, alcohol use, comorbidities, meat intake, meat doneness, activity level, total fat intake, and total fiber intake. Due to our limited sample size, features were carefully selected for inclusion in the logistic regression model to maximize degrees of freedom and reduce variability. Features were selected based on univariate analysis and expert guidance. Models were fitted twice, once with imputed variables and once with patients with missing variables excluded. If data imputation did not significantly alter the results for our variable of interest, choline, then the exclusion method was used.

To study the potential effect modification of choline and the risk of CRC by B vitamins, each B vitamin was separately added to the logistic regression. To test for statistical interaction, we included the cross-product of dietary choline intake and dietary B-vitamin intake for each corresponding B-vitamin model. Interaction terms were removed from the model if not statistically significant. Odds ratios were calculated by exponentiating the coefficient. Because dietary choline intake was transformed via log base 2, the odds are interpreted for every doubling of dietary choline intake rather than every one-unit increase. Additionally, we performed subgroup analyses stratified by high/low B-vitamin intake to examine whether they modified the association between high/low choline intake and CRC. The cutoff values for binary stratification were based on the median value among participants with normal outcomes. Statistical tests were 2-tailed, and *p*-value < 0.05 was considered statistically significant. Analyses were conducted by Python version 3.7.4 with the statsmodels package.

## 3. Results

### 3.1. Study Population Characteristics

Baseline characteristics for the study population are found in Table 1. A total of 52 patients participated in the study, of which 21 had a normal colonoscopy result, defined as the absence of polyps or CRC, and 31 had an abnormal colonoscopy result, defined as the presence of polyps or CRC. Compared to the patients with a normal colonoscopy, patients with an abnormal colonoscopy result were older, non-White, had a higher BMI, participated in fewer weekly minutes of light exercise and lifting, and had a higher percentage with a smoking history, diabetes mellitus, ulcerative colitis, and hemorrhoids. Notably, none of these differences, with the exception of weekly minutes spent lifting, were of statistical significance.

In terms of dietary intake, empirically, the abnormal group had higher levels of energy, total meat, red meat, processed meat, weekly alcoholic drinks, total fat, and fiber intake. Statistically, the abnormal group had higher total meat (*p* = 0.05) and red meat (*p* = 0.03) intake. The abnormal group also had a higher daily intake of all B vitamins, choline, and betaine compared to the normal group.

### 3.2. Correlation of Dietary Choline and Meat Intake

**Figure 3:**
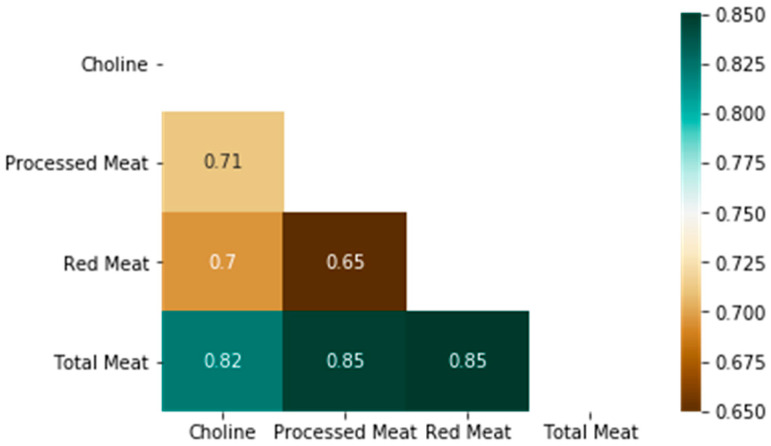
Correlation matrix of dietary choline intake with dietary meat intake.

A heatmap of the correlation matrix between dietary choline and meat intake is shown in Figure 3. Choline is a nutrient abundant in diets rich in animal protein with high-fat content [3]. As expected, dietary choline intake is positively associated with dietary total meat (r = 0.82, *p* < 0.001), red meat (r = 0.70, *p* < 0.001), and processed meat (r = 0.71, *p* < 0.001) intake.

### 3.3. Association of Dietary Choline Intake and Abnormal Colonoscopy Result

Based on univariate analysis, the inclusion of the meat doneness variable resulted in the greatest percent change for the dietary choline coefficient. Based on expert opinion, total energy intake and age were also included in the final model. Imputation of the data did not significantly alter results for our variable of interest, choline. Thus, missing data was handled via exclusion, with a total sample size of 44 (6 patients missing data for choline and energy, 2 patients missing data for meat doneness). Table 2 displays the odds ratios of dietary choline intake as calculated from the logistic regression models, after adjusting for the aforementioned features. Because dietary choline intake was transformed via log base 2, the odds are interpreted for every doubling of dietary choline intake rather than every one-unit increase. A doubling in dietary choline intake was significantly associated with increased odds of polyps or CRC (OR 25.32, 95% CI 1.95–327.94).

### 3.4. Effect Modification by B-Vitamins

The interaction terms represented by the cross-products of dietary choline intake and B vitamins were all not found to be statistically significant. Thus, the interaction terms were not included in the logistic regression models. When handling missing data by exclusion, the inclusion of each B vitamin into the model did not change the overall significance of choline. In other words, dietary choline intake is still associated with greater odds of polyps or CRC after adjusting for dietary B-vitamin intake. However, when handling missing data by imputation to the mean, the inclusion of vitamins B6, B9, and B12 did, in fact, modify the harmful effect of choline. After independently adjusting for dietary vitamin B6, B9, and B12 intake, dietary choline intake was not found to have a statistical association with abnormal colonoscopy results, suggesting that dietary B-vitamin intake may counteract the harmful effects of choline. To further analyze this effect modification, each B vitamin was stratified based on the median value among the normal group. Within each high/low B-vitamin stratification, the exposure of high dietary choline intake was assessed compared to low dietary choline intake. Observationally, there were more abnormal colonoscopy outcomes than normal in the high dietary choline group, as shown in Table 3. However, due to the limited sample size, the significance of the calculated odds ratios is difficult to interpret.

## 4. Discussion

This case-control study investigates the association between dietary choline intake and colorectal polyp and CRC outcomes and examines the potential modifying effects of B vitamins, particularly vitamins B2, B6, B9, and B12. Our analysis revealed a significant positive association between higher dietary choline intake and abnormal colonoscopy results, indicating the presence of polyps or CRC, even after adjusting for various confounding factors. Importantly, our results suggest that adequate intake of dietary B vitamins, notably vitamin B12, may attenuate the harmful effects associated with high choline intake on CRC risk. This potential protective effect of B vitamins against CRC risk, particularly in the context of diets high in red and processed meat, merits deeper exploration of the biochemical mechanisms involved.

Red and processed meats, typical of Western diets, are rich in multiple nutrients such as choline [21,22], iron [23,24,25,26], zinc [24,25], vitamin B12 [25,26], and carnitine [27], which participate in various metabolic pathways that could either promote or protect against cancer, depending on the context and balance of intake. Choline is a precursor for several biochemical processes, including the synthesis of phosphatidylcholine, acetylcholine, and methyl groups involved in DNA methylation [28]. When oxidized to betaine, choline plays an integral role in one-carbon metabolism [29,30], which is essential for DNA synthesis, repair, and methylation—all of which are crucial in maintaining genomic stability and preventing malignant transformation. However, choline also serves as a substrate for gut microbiota, which can convert it into trimethylamine (TMA), subsequently oxidized to trimethylamine N-oxide (TMAO) in the liver [31,32,33]. TMAO has been implicated in inflammatory processes that could contribute to the development of CRC by promoting cellular proliferation, angiogenesis, and a pro-inflammatory environment in the colon [34,35].

In this study, we observed a significant association between high choline intake and CRC risk, aligning with the hypothesis that excessive dietary choline from red meat could increase carcinogenesis risk through multiple pathways. The strong correlation observed between choline intake and red meat intake suggests that individuals consuming large quantities of red meat inherently consume more choline. In high-red-meat diets, elevated choline levels may facilitate TMA production by the gut microbiota, subsequently increasing TMAO levels. This increase could lead to a pro-inflammatory state that promotes CRC by inducing cellular dysplasia, proliferation, and potential malignant transformation.

Vitamin B12, which is also abundant in red meat, plays a critical role in one-carbon metabolism and heme synthesis [36]. Adequate levels of B12 and other B vitamins are necessary cofactors in one-carbon metabolism, enabling choline to be used for DNA synthesis and repair rather than TMA production [37,38]. In this metabolic pathway, B vitamins stabilize DNA, promote methylation, and maintain genomic integrity, all of which can reduce CRC risk. However, when B-vitamin levels are insufficient, particularly vitamin B12, choline may be diverted toward alternative metabolic pathways, increasing the production of TMA and subsequently TMAO, promoting inflammation and CRC risk [3,10]. This aligns with Le Chatelier’s principle [39], where a lack of cofactors (such as B vitamins) shifts metabolic reactions toward other pathways—in this case, favoring TMAO production over one-carbon metabolism.

The interplay between choline and B vitamins has been previously studied with mixed results. Some research suggests that B-vitamin deficiency might prompt choline to act as a compensatory methyl donor [36,40], theoretically protecting against carcinogenesis. However, this hypothesis does not consider the impact of TMAO production in a high-choline, low-B-vitamin diet, particularly one dominated by red meat. In our findings, B-vitamins, especially vitamin B12, appeared to mitigate the risk of CRC associated with choline intake, possibly by directing choline toward DNA synthesis and repair rather than TMAO production. This suggests that individuals with low B-vitamin intake may be at greater risk for CRC when consuming a diet rich in choline and red meat, as choline metabolism may be diverted toward TMAO, a known pro-inflammatory compound associated with CRC.

The gut microbiota also plays a significant role in how dietary choline is metabolized. Studies indicate that specific bacterial populations in the gut can convert choline into TMA, which is subsequently oxidized into TMAO in the liver [3,31,41]. The extent of TMA production may vary between individuals due to differences in microbiota composition, which may predispose certain people to a higher CRC risk from red meat and choline-rich diets. It is unclear if a high-choline diet induces dysbiosis, encouraging the growth of TMA-producing bacteria, or if individuals with existing dysbiosis are more susceptible to CRC due to higher TMA production. Investigating how dietary choline influences gut microbiota composition and whether B-vitamin supplementation affects the microbial conversion of choline could provide valuable insights into CRC prevention strategies.

Our study has several limitations, primarily due to the relatively small sample size, which constrains our ability to draw definitive conclusions, particularly in stratified analyses. This limits the statistical power to detect nuanced interactions between choline, B vitamins, and CRC risk. Nevertheless, our single-site, geographically focused cohort provides insights into these interactions in the context of a Western dietary pattern high in red and processed meat. This design reduces variability in dietary patterns and strengthens the internal validity of our findings, though it may limit generalizability. Given the complexity of the interactions between choline, B vitamins, and CRC risk, further research with larger and more diverse populations is essential to validate and expand on our findings.

## 5. Conclusions

In this study, we observed a significant association between high dietary choline intake, particularly from red and processed meats, and an increased risk of colorectal polyps and CRC. Our findings suggest that adequate intake of B vitamins, especially vitamin B12, may attenuate this risk by supporting one-carbon metabolism pathways that contribute to DNA synthesis, repair, and methylation, thereby reducing genomic instability and cancer risk. Conversely, insufficient B-vitamin intake may shift choline metabolism toward trimethylamine (TMA) and trimethylamine N-oxide (TMAO) production, compounds linked to inflammation and CRC promotion.

Our results highlight the importance of considering both choline and B-vitamin intake levels, especially in Western populations with high red meat consumption. Additionally, the role of gut microbiota in choline metabolism and TMAO production represents a key area for future research, as variations in microbial composition may influence individual CRC risk associated with high-choline diets.

These findings underscore the potential of B-vitamin supplementation and dietary modifications as preventive strategies for CRC, particularly in individuals at elevated risk due to dietary habits. Further research with larger, more diverse populations is needed to confirm these findings and develop targeted dietary guidelines and interventions for reducing CRC risk.

## Figures and Tables

**Table 1:** Study subject baseline characteristics.

	Normal (n = 21)	Abnormal (n = 31)	Overall (n = 52)	Missing Data	*p*-Value ^1^
**Age, mean (SD)**	59.6 (5.8)	61.8 (8.2)	60.9 (7.4)	0	0.26
**Sex, female, n (%)**	12 (57.1)	18 (58.1)	30 (57.7)	0	1.00
**Ethnicity, n (%)**				0	0.63
White	14 (66.7)	18 (58.1)	32 (61.5)		
Black	7 (33.3)	12 (38.7)	19 (36.5)		
Black Hispanic	0 (0)	1 (3.2)	1 (1.9)		
**Smoking, n (%)**				1	0.38
Nonsmoker	11 (52.4)	10 (33.3)	21 (41.2)		
Past smoker	6 (28.6)	13 (43.3)	19 (37.3)		
Current smoker	4 (19.0)	7 (23.3)	11 (21.6)		
**Family history of CRC**	5 (26.3)	6 (23.1)	11 (24.4)	0	1.00
**BMI (kg/m^2^), mean (SD)**	30.9 (6.5)	32.3 (6.1)	31.7 (6.2)	2	0.44
**Comorbidities, n (%)**					
Diabetes Mellitus	5 (23.8)	9 (30.0)	14 (27.5)	0	0.87
Crohn’s disease	0 (0)	0 (0)	0 (0)	1	1.00
Ulcerative colitis	0 (0)	1 (3.3)	1 (2.0)	0	1.00
Hemorrhoids	3 (14.3)	11 (36.7)	14 (27.5)	0	0.15
Anal tears	1 (4.8)	1 (3.3)	2 (3.9)	0	1.00
Peptic ulcer	1 (4.8)		1 (2.0)	0	0.41
Diverticulitis	6 (28.6)	7 (23.3)	13 (25.5)	0	0.92
Gastroenteritis	1 (4.8)	1 (3.3)	2 (3.9)	0	1.00
Irritable bowel syndrome	4 (19.0)	2 (6.7)	6 (11.8)	0	0.21
Angiodysplasia	21 (100.0)	30 (100.0)	51 (100.0)	1	1.00
**Physical Activity (min/wk), mean (SD)**					
Walking	41.6 (71.6)	41.9 (80.7)	41.8 (76.5)	0	0.91
Lifting	25.3 (45.5)	6.9 (22.9)	14.4 (34.7)	0	0.04
Light exercise	98.1 (116.4)	76.6 (102.4)	85.3 (107.7)	0	0.50
Moderate exercise	93.5 (100.1)	92.5 (109.4)	92.9 (104.8)	0	0.97
Vigorous exercise	43.9 (76.7)	39.6 (79.0)	41.4 (77.4)	0	0.24
**Dietary intake, mean (SD)**					
Energy (kcal/day)	1391.8 (938.9)	1757.6 (920.1)	1630.4 (933.0)	6	0.16
Total meat (oz/day)	2.1 (2.0)	3.3 (2.3)	2.9 (2.3)	6	0.05
Red meat (oz/day)	0.7 (0.8)	1.2 (0.8)	1.0 (0.8)	6	0.03
Processed meat (oz/day)	0.6 (0.7)	0.9 (0.9)	0.8 (0.8)	6	0.18
Meat doneness (steak, burger) ^2^	2.4 (0.9)	2.7 (0.7)	2.6 (0.8)	2	0.17
Alcohol (g/day)	17.9 (49.4)	8.6 (26.0)	11.9 (35.6)	6	0.73
Alcohol (standard drinks/wk)	8.3 (18.6)	10.9 (33.9)	9.9 (28.4)	3	0.87
Total fat (g/day)	52.2 (42.3)	64.1 (33.1)	60.0 (36.5)	6	0.11
Total fiber (g/day)	13.5 (10.8)	16.0 (9.7)	15.1 (10.0)	6	0.21
Vitamin B2 (mg/day)	1.6 (0.9)	1.9 (1.1)	1.8 (1.0)	6	0.26
Vitamin B6 (mg/day)	1.3 (0.9)	2.0 (1.3)	1.7 (1.2)	6	0.06
Vitamin B9 (mcg/day)	244.3 (174.0)	324.6 (172.2)	296.7 (175.2)	6	0.11
Vitamin B12 (mcg/day)	2.9 (1.9)	4.7 (3.5)	4.1 (3.1)	6	0.05
Choline (mg/day)	207.0 (130.6)	297.5 (167.8)	266.0 (160.4)	6	0.06
Betaine (mg/day)	70.2 (56.8)	111.8 (76.0)	97.4 (72.1)	6	0.04

Note: ^1^. Difference between subjects with normal and abnormal colonoscopy; ^2^. Meat doneness self-reported on a scale of 0–4.5 based on a set of images. CRC, colorectal cancer; BMI, body mass index.

**Table 2:** Odds ratio of abnormal colonoscopy results after doubling dietary choline intake adjusted by vitamin B intake.

	Odds Ratio [95% CI]	*p*-Value
Choline	25.32 [1.95, 327.94]	0.013
Choline + B2	33.13 [2.36, 465.34]	0.009
Choline + B6	23.06 [1.76, 301.72]	0.017
Choline + B9	21.87 [1.60, 298.10]	0.021
Choline + B12	53.64 [2.50, 1152.39]	0.011

Logistic regression model equation: abnormal outcome~meat doneness + age + total energy intake + dietary choline intake ± dietary B vitamin intake.

**Table 3:** Odds ratio of abnormal colonoscopy results according to dietary choline intake stratified by vitamin B intake.

	Choline < 252.94 mg		Choline ≥ 252.94 mg		OR (95% CI)
	Abnormal	Normal	Abnormal	Normal	
B2 < 1.64 mg/day	12	7	4	1	2.33 [0.22, 25.25]
B2 ≥ 1.64 mg/day	1	1	13	7	1.86 [0.10, 34.44]
B6 < 1.14 mg/day	5	7	1	1	1.40 [0.07, 28.12]
B6 ≥ 1.14 mg/day	8	1	16	7	0.286 [0.03, 2.74]
B9 < 195.43 mcg/day	5	7	0	1	0.700 [0.02, 25.20]
B9 ≥ 195.43 mcg/day	8	1	17	7	0.30 [0.03, 2.90]
B12 < 2.61 mcg/day	9	8	0	0	-
B12 ≥ 2.61 mcg/day	4	0	17	8	0.27 [0.01, 5.65]

## Data Availability

The data presented in this study are available on request from the corresponding author. The data are not publicly available due to privacy restrictions.
